# Frizzled7: A Promising Achilles’ Heel for Targeting the Wnt Receptor Complex to Treat Cancer

**DOI:** 10.3390/cancers8050050

**Published:** 2016-05-17

**Authors:** Toby Phesse, Dustin Flanagan, Elizabeth Vincan

**Affiliations:** 1 Molecular Oncology Laboratory, Victorian Infectious Diseases Reference Laboratory and the Doherty Institute, University of Melbourne, Melbourne, VIC 3000, Australia; dustin.flanagan@unimelb.edu.au; 2 Walter and Eliza Hall Institute of Medical Research, Melbourne, VIC 3052, Australia; 3 School of Biomedical Sciences, Curtin University, Perth, WA 6102, Australia

**Keywords:** Wnt, cancer, Frizzled, Frizzled7, FZD7, Fz7, therapy, cell signalling, receptor, PCP

## Abstract

Frizzled7 is arguably the most studied member of the Frizzled family, which are the cognate Wnt receptors. Frizzled7 is highly conserved through evolution, from *Hydra* through to humans, and is expressed in diverse organisms, tissues and human disease contexts. Frizzled receptors can homo- or hetero-polymerise and associate with several co-receptors to transmit Wnt signalling. Notably, Frizzled7 can transmit signalling via multiple Wnt transduction pathways and bind to several different Wnt ligands, Frizzled receptors and co-receptors. These promiscuous binding and functional properties are thought to underlie the pivotal role Frizzled7 plays in embryonic developmental and stem cell function. Recent studies have identified that Frizzled7 is upregulated in diverse human cancers, and promotes proliferation, progression and invasion, and orchestrates cellular transitions that underscore cancer metastasis. Importantly, Frizzled7 is able to regulate Wnt signalling activity even in cancer cells which have mutations to down-stream signal transducers. In this review we discuss the various aspects of Frizzled7 signalling and function, and the implications these have for therapeutic targeting of Frizzled7 in cancer.

## 1. Introduction

Frizzled7 (FZD7) is located on human chromosome 2q33 and contains 3859 nucleotides that produce a 574 amino acid transmembrane protein, which belongs to a family of Wnt receptors. The Wnt signalling pathway regulates a diverse repertoire of cellular functions, playing critical roles during embryonic development, stem cell function and is often deregulated in cancer. Frizzled (Fzd) receptors have an extracellular N-terminal with a conserved cysteine rich domain (CRD), which is responsible for binding to Wnt ligands, seven transmembrane domains and an intracellular C-terminal with a putative PDZ binding domain. The number of Fzd genes varies between class of organism; mammals have ten Fzd’s, *Drosophila* have four, whilst *C. elegans* have three. Sequence analysis indicates that vertebrate Fzd genes can be divided into discrete classes based on structural homology. Fzd1, Fzd2 and Fzd7 share approximately 97% identity, Fzd5 and Fzd8 share 70% identity, Fzd4, Fzd9 and Fzd10 share 65% identity and Fzd3 and Fzd6 share 50% amino acid identity [1,2]. Fzd receptors are able to associate with several distinct co-receptors giving the Wnt pathway considerable complexity and flexibility. The cytoplasmic components of Wnt signalling are frequently mutated in cancer, for example ~90% of colorectal cancers contain mutations which activate the Wnt pathway [3]. However, it is becoming clear that the Wnt receptor complex also plays an important role during cancer despite mutations in the downstream components of the pathway. There is now convincing data to demonstrate that the level at which the Wnt pathway is activated is instrumental to phenotypic outcomes, and that the strength of the signal is regulated at multiple points in the signalling cascade including the Wnt/Fzd receptor complex. Fzd7 is of particular interest as it is the Wnt receptor most commonly up-regulated in several different cancers and can also transmit signals through the different arms of the Wnt pathway referred to broadly as canonical (Wnt/β-catenin dependent) and non-canonical (Wnt/β-catenin independent). The decision of which pathway Fzd7 will signal through upon ligand binding is still a subject of current research in the field, but appears to be dependent on the availability of co-receptors, Wnts and the cellular and extra-cellular milieu. As both the canonical and non-canonical pathways are deregulated in many different cancers, Fzd7 offers an exciting target for therapeutic intervention and will be the main focus of this review.

## 2. Wnt Signalling

The Wnt1 gene (originally called Int1) was first identified during a screen to identify oncogenes capable of inducing breast tumours in mice when mutated by infection with mouse mammary tumour virus (MMTV) [4]. Since then, a whole family of Wnt proteins has been discovered (19 in mammals, including humans) which are highly conserved from humans through to the simplest free living organisms, placozoans [5] and sponges [6]. Experiments in *Drosophila* and *Xenopus* demonstrate that Wnt1 is an important regulator of many cell functions during development including proliferation, migration, differentiation, apoptosis and cell polarity. These experiments, and subsequent ones, identified that Wnt1 is a homolog of *Drosophila Wingless (Wg)* and is the ligand for a signalling pathway which signals through Glycogen synthase kinase-3 beta (GSK3β), β-catenin and the transcription factors T-cell factor-4 (Tcf4)/Lymphoid enhancer binding factor-1 (Lef1), and was consequently called the Wnt pathway (an amalgam of “Wg” and “Int1”) [7]. In addition to its critical role during development, the Wnt pathway also controls the activity of stem cells in adult tissues including the intestine, stomach, liver, ovary, breast and hair follicle, during homeostasis and regeneration [8].

Wnt proteins are ~40 KDa in size and mediate relatively close-range signalling between cells. Recent evidence for the mechanism of this short-range signalling suggests that lipid modifications to Wnt by the palmitoyltransferase enzyme Porcupine in the endoplasmic reticulum target it for tethering to the membrane by the carrier protein Wntless [9]. Thus exocytosed Wnt proteins are thought to be either attached to the cell membrane or expelled in secretory vesicles, with very recent evidence suggesting that cell division, and not diffusion, is the primary mechanism for forming Wnt gradients, at least in the mammalian intestinal crypt [10]. However, studies in *Drosophila* suggest Wg can act as both a short and long-range inducer, which presents an interesting paradox; how do Wg ligands, which are hydrophobic and prone to form aggregates in an aqueous extracellular environment, act as long-range inducers if their lipid moieties confer high affinity for membrane tethering? Recent research from Roel Nusse’s lab has addressed this long standing question. Secreted Wg-interacting molecule (Swim), a member of the Lipocalin family of transporter proteins, can bind with nanomolar affinity to Wg, chaperoning Wg through the extracellular milieu, whilst preserving Wg solubility and signalling activity [11]. As such, RNAi-mediated *Swim* knockdown disrupts the identity of cells positioned distally from the local Wg source, demonstrating that they require long-range Wg signaling. As anticipated, *Swim* reduction resembles *Wg* mutant long-range phenotypes [11]. Furthermore, seminal work from Vivian Budnik’s group has elegantly demonstrated in the *Drosophila* neuromuscular junction (NMJ) that Wg signal transmission can occur via exosome release, which contain the Wnt binding protein Evenness Interrupted (Evi) [12]. Evi is required for Wg secretion on the pre-synaptic side, as *Evi* mutants phenocopy *Wg* mutant tissues [13]. Additionally, Evi plays an important cell autonomous role targeting dGRIP, a Wg-receptor-interacting protein, to post-synaptic sites where it facilitates post-synaptic Wg signal transmission [13]. Besides its role in transporting Wg via exosomes across the NMJ, Evi also functions in the retromer system, where it is recycled from endosome to the Golgi apparatus for subsequent rounds of Wg release at the plasma membrane [12].

## 3. Canonical and Non-Canonical Signalling

**Figure 1:**
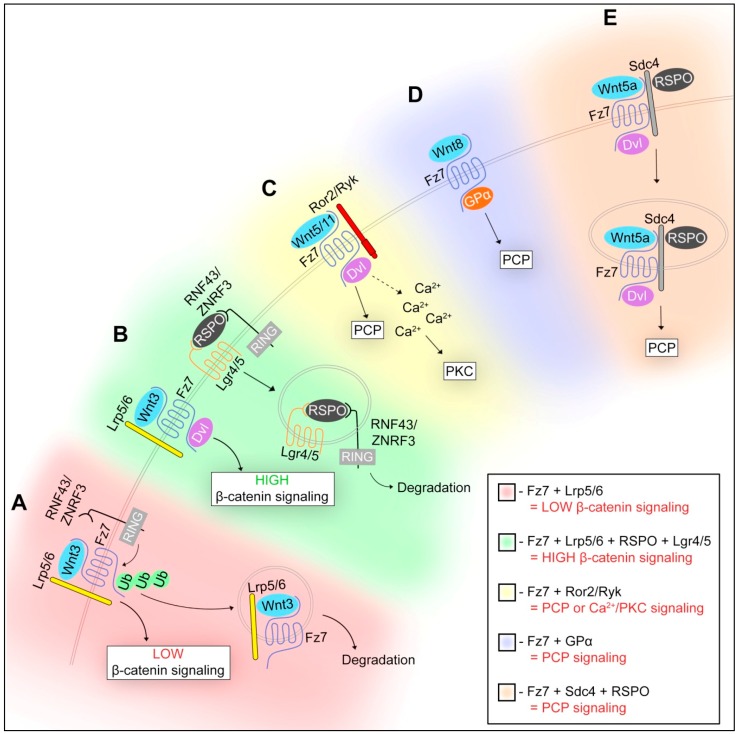
Fzd7 receptor complexes and signalling output. (**A**) Fzd7 (Fz7) can associate with Lrp5/6 to transmit canonical Wnt signaling from ligands including Wnt3. However RNF43/ZNRF3 ubiquitylates Fzd7 and targets it for internalisation and protesomal degradation, thus resulting in turn-over of the receptor complex and low canonical Wnt signaling; (**B**) In the presence of Lgr4/5, and RSPO it is RNF43/ZNRF3 which is internalised and degraded and thus Fzd7/Lrp5/6 remains on the cell surface to transmit Wnt signalling and canonical signalling output is high; (**C**) Fzd7 can associate with Ror2 or Ryk to transmit non-canonical Wnt signals from ligands including Wnt5 or Wnt 11, either via PCP or Ca^2+^; (**D**) Fzd7 can associate with G Protein α (GP α) to transmit signals from Wnt8 via the PCP pathway; (**E**) Fzd7 can associate with Syndican4 (Sdc4) and R-Spo to transmit Wnt5a signals via internalisation of the whole receptor complex and ligand which then activates PCP signaling.

Wnt signalling is broadly divided into canonical and non-canonical pathways based primarily on whether signalling activates β-catenin (for canonical), and associated phenotypes including transformation of mammary epithelial cells and *Xenopus* axis duplication for canonical signalling and orientation of hairs on the wing surface of *Drosophila* for non-canonical. The interaction between the Wnt ligand and the receptors it associates with on neighbouring cells determines which of these pathways is activated (Figure 1). Some Wnts that have been shown over many years of research to robustly activate particular pathways, such as Wnt3a and Wnt2b activating canonical signalling whilst Wnt5a and Wnt11 predominantly activate non-canonical pathways, however, this is by no means an exclusive activation of just one pathway. Indeed, several studies now indicate considerable cross-talk between the canonical and non-canonical pathways and thus this division should not been considered entirely separate but rather as a signalling network depending on cell and situation context [14]. Consequently, Wnt5a provides an excellent example of how a single Wnt ligand can activate discrete signalling outcomes, depending on cellular context and receptor availability. Well studied for its role in PCP and Wnt/Ca^2+^ activation (discussed in detail below), Wnt5a in co-operation with Ror2 can inhibit Wnt3a-mediated canonical Wnt signalling through promoting β-catenin degradation independent of Gsk-3 [15,16]. Demonstrated in parallel, Wnt5a/Ca^2+^ signalling triggers calcium calmodulin dependent protein kinase II (CaMKII) activation, which induces the Transforming growth factor-beta-activated kinase 1 (TAK1)-Nemo-like kinase (NLK)-Mitogen-activated-protein-kinase (MAPK) pathway, shown to phosphorylate TCF/LEF factors and inhibit the interaction of the β-catenin/TCF complex with DNA. Thus, the TAK1-NLK-MAPK-like pathway, via Wnt5a/Ca^2+^ signaling, negatively regulates the Wnt/β-catenin signalling pathway [17,18]. Interestingly, in other contexts, Wnt5a can activate β-catenin/TCF/LEF-mediated transcription in the presence of the appropriate Fzd receptor, Fzd4 [15].

The pivotal effector of the canonical Wnt pathway is the co-transcription factor β-catenin. In the absence of active Wnt signalling, β-catenin is primarily localised to cell-cell adherens junctions and any free cytoplasmic β-catenin is rapidly phosphorylated by GSK3 in a degradation complex which targets β-catenin for proteosomal degradation. The destruction complex also includes Adenamatous polyposis coli (Apc), Casein kinase 1 (CK1) and Axin to facilitate phosphorylation of β-catenin. During canonical Wnt signaling, Wnt ligands bind to transmembrane Fzd receptors and low-density lipoprotein receptor-related protein 5 or 6 (Lrp5/6) co-receptors. Once activated, this receptor complex associates with the cytosolic scaffold protein Dishevelled (Dvl) to inhibit GSK3 enzyme activity. Consequently, β-catenin escapes phosphorylation and subsequent degradation, accumulates in the cytoplasm and translocates into the nucleus. β-catenin cannot bind to DNA directly but instead regulates transcription by associating with the transcription factor complex Tcf4/Lef1 that bind to conserved E-box sequences in regulatory elements of genes. Wnt1 and Wnt3a are prototypical examples of ligands that consistently activate this pathway [19].

The non-canonical Wnt pathway is defined as being able to signal independent of β-catenin and has two arms; the Planar Cell Polarity (PCP) pathway and the Calcium pathway (Ca^2+^). PCP signalling is activated when Wnt ligands including Wnt5a [20], Wnt7a [21], Wnt8b [22] or Wnt11 [23,24] bind to Fzd receptors and associated receptor tyrosine kinases such as Ror2 or Ryk [25], thus allowing for significant complexity in the transmission of the signal. Depending on which Wnt/receptor complex is activated, a variety of cytoplasmic PCP pathway signal transduction components are then activated including DVL, Cdc42, c-JUN N-terminal Kinase (Jnk) and the GTPases RhoA, Rac1 [26]. The PCP pathway also includes the so called “core” components (Fzd, Dsh, Celsr, Vangl and Prickle) which set up cell polarity within the plane of the epithelial sheet [27]. Although primarily involved in cell polarity and migration, recent data suggests that PCP signalling can also promote proliferation in several different cancers [28,29,30,31,32].

The Wnt/Ca^2+^ pathway derives originally from the observations that over-expression of Wnt5a or rat Fzd2 can cause an increase in intracellular calcium in zebrafish [33] and can activate protein kinase C (PKC) and calcium/calmodulin-dependent protein kinase (CaM kinase) in *Xenopus* [34]. Wnt/Fzd ligand receptor interaction with participating co-receptor Ror 1/2 leads to the production of inositol 1,4,5-triphosphate (IP3) and 1,2 diacylglycerol (DAG) and calcium from membrane-bound phospholipid phosphatidyl inositol 4,5-bisphosphate via the action of membrane-bound enzyme phospholipase C (PLC). PLC then modifies IP3 and DAG allowing IP3 to diffuse through the cytosol and interact with calcium channels present on the endoplasmic reticulum, which result in the release of calcium ions. Calcium in conjunction with calmodulin activates CaMKII. Meanwhile, DAG and intracellular calcium activate PKC. PKC and CaMKII can activate various regulatory proteins, including NFκB and CREB, which are nuclear transcription factors, and thus regulate target gene expression [35].

## 4. Regulators of Wnt/Fzd Receptor Interaction

In canonical Wnt signalling, Fzd receptors respond to Wnt proteins in the presence of co-receptor LRP5/6 to activate and propagate the canonical Wnt/β-catenin pathway. Alternatively, Fzd receptors can respond to Wnt proteins in the presence of the Wnt co-receptor Ror2 to activate the non-canonical pathway [20]. Thus, it seems likely that the decision for a cell to activate canonical or non-canonical Wnt signalling largely depends on the receptor/co-receptor/ligand combination and availability of these components, which is a current research focus in the field (Table 1 and Figure 1) [14]. This interaction between Wnt ligand and its co-receptors is also regulated by several other proteins, to ensure that Wnt signalling is tightly regulated as described below.

### 4.1. sFRP Protein Family

The family of secreted frizzled-related proteins (sFRPs) is comprised of five secreted glycoproteins (sFRP-1, sFRP-2, sFRP-3, sFRP-4, and sFRP-5), which bind directly to Wnts, thereby altering their ability to bind to the Wnt receptor complex [36]. All sFRP family members contain an N-terminal domain that is 30%–50% homologous to the CRD of Frizzled receptors, which is considered both necessary and sufficient for Wnt binding and inhibition [37,38]. In contrast to Fzd family proteins, sFRPs lack a transmembrane region and the cytoplasmic domain required for signal transduction into the cells. However, other structure and function analyses of sFRP1 demonstrate that both the CRD and C-terminal Netrin-related motif (NTR) domains are required for optimal Wnt inhibition [39].

The crystal structures of the CRDs from mouse Fzd8 and sFRP3 reveal the potential to form dimers [40], which is supported by evidence showing Fzd and sFRP proteins can form homo- and heteromeric complexes via the CRD [41]. These findings may imply that sFRPs have multiple modes in which they can inhibit Wnt signalling. Unlike Dkks, which specifically target and inhibit canonical Wnt signalling, sFRPs can bind directly to Wnt ligands, thereby inhibiting both canonical and non-canonical Wnt signalling [36,42].

It should be noted that sFRPs do not always inhibit Wnt signalling, as nanomolar concentrations of sFRP1 increase Wnt3a signalling in HEK293 cells, whilst higher concentrations inhibit Wnt signalling [43]. In addition to the concentration specific activities of sFRPs [44], cell type and the expression of Fzd receptors [43] and GSK3 [45] have also been show to modulate the ability of SFRPs to inhibit Wnt signalling. For example, sFRP2 has been shown to inhibit Wnt3a signalling in ES cells [44], whilst in HEK293 cells it inhibits Wnt signalling [46].

### 4.2. Wnt Inhibitory Factor 1 (WIF-1)

Similar to SFRPs, Wnt inhibitory factor 1 (WIF-1) is also able to bind to Wnt ligands and inhibit Wnt signalling [47]. WIF-1 is conserved amongst the vertebrates and consists of an N-terminal secretion signal sequence, the WIF domain, five Epidermal growth factor (EGF)-like domains and a hydrophilic C terminus [48]. Structural analysis, site mutagenesis and cell assays show that Wnt can bind to the WIF domain and the EGF-like domains of WIF-1 [48].

WIF-1 inhibits Wnt signalling during development to help regulate Wnt expression in the paraxial mesoderm [47], and its promoter region is frequently methylated, and consequently downregulated in many cancers as detailed below.

### 4.3. Dkk Protein Family

There are four Dickkopf (Dkk) proteins in the vertebrate genome, Dkk-1, -2, -3 and -4. Dkk proteins selectively antagonise canonical Wnt signalling by binding to Lrp5/6, thus preventing Fzd and Wnt to form a ternary complex [49]. They can also modulate Wnt signalling by associating with Kremen1 and 2 to form a complex which regulates internalisation of Lrp [50]. Dkks are critical during development, and forced expression of Dkk1 in the mouse intestine inhibits epithelial cell proliferation and results in a loss of stem cells, highlighting the importance of Wnt signalling in intestinal stem cells [51,52].

### 4.4. R-Spondin & the E3 Ligases (Rnf43 & Znrf3)

R-Spondin (R-Spo) proteins are potent agonists of Wnt signalling, but only in the presence of Wnt ligands [53]. There are four R-Spo proteins in the vertebrate genome, R-Spo1, 2, 3 and 4. These excreted proteins are characterised by two N-terminal furin domains, a thrombospondin domain in the centre of the protein and a positively charged C-terminal region [54]. Gene expression and co-immunoprecipitation analysis revealed that Lgr proteins form a complex with other Wnt receptor components including Fzd and Lrp5/6, suggesting a potential role as a Wnt receptor [55,56]. It was subsequently shown that tagged R-Spo1 can bind to Lgr4, -5, and -6 in HEK293 and Ls174 cells. In support of an interaction between R-Spo and Lgr, R-Spo/Lgr complexes are revoked following Lgr4 or 5 deletions [55,56].

The highly related transmembrane E3 ubiquitin ligases, ZNRF3 and RNF43, act as negative regulators of canonical Wnt signalling that are integral to R-Spo/Lgr Wnt potentiation [57,58]. Both ZNRF3 and RNF43 were identified from gene expression profiling to discover genes that positively correlated with known negative regulators of Wnt signalling, such as Axin2 or Dkk-1 [57]. In common with other Wnt antagonists, including Axin2 and Dkk, ZNR43 and RNF43 are also Wnt target genes [57]. ZNRF3 regulates Wnt signalling by ubiquitylating Fzd-Lrp receptor complexes and thus targeting them for internalisation and proteasomal degradation [57]. It has also been demonstrated that R-Spo can induce membrane clearance of ZNRF3 through ZNRF3-Lgr4 dimerisation, thus leading to an accumulation of Wnt receptors on the cell surface [57,59]. These findings support a model where in the absence of R-Spo, ZNRF3 ubiquitylates Fzd and promotes the degradation of Fzd-LRP6 complex, thus keeping Wnt signalling to low levels. However, in the presence of R-Spo, an interaction between Lgr4 and ZNRF3, via R-Spo Furin domains (1 & 2), leads to the clearance of ZNRF3, allowing Fzd/LRP6 to stabilise and accumulate at the membrane to enhance canonical Wnt signalling [57,59] (Figure 1).

In addition to its important role in regulating canonical Wnt signalling, R-Spo3, is able to regulate the Wnt/PCP pathway [56]. R-Spo3 forms a complex with Fzd7 to transmit Wnt5a signals by inducing Sdc4-dependent, clathrin-mediated endocytosis during development (Table 1 and Figure 1). These data support a model whereby internalisation of the Wnt/receptor complex is an important mechanism by which R-Spondins promote Wnt/PCP signalling [60].

## 5. The Wnt Receptor as a Therapeutic Target in Cancer

A vast body of research data exists on the cytoplasmic events involved in Wnt signalling [72] but only relatively recently have the Wnt receptors that transmit these signals began to attract more interest. This is due, in part, to the observations made in the 1990’s that the cytoplasmic components of Wnt signalling are often mutated in many different cancers, thus resulting in more intensive research into their function and potential as targets for therapeutic intervention. For example, it is now established that *Apc* is mutated in 80%–90% of colorectal tumours [73]. As such, strategies to target the Wnt receptor complex in cancers harbouring mutations to downstream Wnt signalling components, e.g., Apc in colorectal tumours, appeared counter-intuitive. However, in 2002, observations from Ron Smits lab based on the location of mutations occurring naturally in the *Apc* gene led them to propose the “just right” or Goldilocks model of Wnt signalling in cancer which proposes an optimal, but not excessive, level of Wnt pathway activation to transform cells, suggestive of constrained Wnt activation [74]. Furthermore, a surprising result from Ian Tomlinson’s group also supported the Goldilocks model, in which a novel Apc mutant mouse (Apc1322T) developed severe intestinal polyposis associated with weaker Wnt pathway activation when compared to *Apc^Min/+^* mice (which are predisposed to develop intestinal tumours after loss of heterozygosity results in *Apc* mutations on both alleles [75,76]. Conversely, work from Alan Clarke’s lab demonstrated that *Apc^Min^* mice deficient for the Wnt target gene *Cited1* developed significantly less tumours and had extended life span compared to their *Apc^Min^*, *Cited1* proficient littermates [77]. Further analysis of this phenotype revealed that *Cited1* deficient mice displayed elevated levels of Wnt signalling during tumourigenesis, which became cytotoxic and induced cell death, consequently resulting in the development of fewer intestinal tumours [77]. This is consistent with many *in vitro* studies demonstrating that high levels of Wnt can induce cell death, but was the first to observe this phenotype during tumourigenesis *in vivo*. Conversely, co-deletion of the Wnt target gene *c-Myc* and *Apc* is sufficient to inhibit all intestinal tumourigenic phenotypes, which is due to restoration of Wnt signaling back to wild type levels. [78]. This concept, that Wnt signalling is not 100% activated when *Apc* is mutated in cancer cells, reveals a therapeutic window whereby targeting the Wnt receptor can still reduce the level of Wnt signalling even in cells which have mutations in *Apc* [78].

The Goldilocks model also requires that the degradation complex is still partially functional even in cells with mutations in *Apc* (a critical component of the complex that phosphorylates β-catenin and targets it for degradation), and thus leaves open the possibility that Wnt signalling can be modified in cells with *Apc* mutations. Indeed, several publications in the mid 2000’s demonstrated that manipulation of factors up-stream of the degradation complex, at the level of the Wnt ligand and receptor, could alter the response of colorectal cancer cells even if they contained mutations to *APC*. For example, the promoter regions of SFRPs [79,80,81] and WIF1 [82] are hypermethylated and consequently down-regulated in the very early stages of gastrointestinal cancer, with the methylation status of certain SFRPs serving as biomarkers for cancer detection and progression [14,83]. The Fzd7 extracellular domain has also been shown to potently inhibit the growth of *APC* mutant CRC tumour cells in a mouse xenograft model [84]. Furthermore, exogenous Wnt originating from tumour associated myofibroblasts was not only capable of maintaining cancer stem cell function and clonality in cells isolated from human colon tumours, but was also able to promote the stemness and clonality of non-cancer stem cells [85]. Importantly, these data indicate that the Wnt receptor complex is not redundant in cancers that have mutations to cytoplasmic Wnt regulators, but in fact still play an active role to promote cancer initiation and growth. As detailed below, many cancers in addition to gastrointestinal cancer have now been reported to display hypermethlylation and downregulation of Wnt ligand/receptor inhibitors such as the SFRP, DKK and WIF, and many also display elevated levels of FZD receptors, suggesting that activation of Wnt signalling via the receptor is involved in driving cancer growth. Furthermore, expression of Wnt and FZD’s are often up-regulated in diverse cancers as detailed below [79,86,87,88].

## 6. Frizzled 7

The Fzd genes were originally discovered along with several other genes including dishevelled (dsh), fuzzy (fy) and multiple wing hairs (mwh), that affect planar cell polarity in *Drosophila*, which was monitored by changes to the orientation of hairs on the wings surface. As such mutations in Fzd genes resulted in changes to the PCP pathway, which disrupted the orientation of wing hairs into a whorl-like appearance [89,90]. Since then, Fzd genes have been found in a diverse range of organisms, from sponges [91] and hydra [92] to vertebrates [93], and have been experimentally demonstrated to be receptors for Wnt ligands [61,94]. Human FZD7 was first identified and characterised in 1998 [1], and was also discovered to be upregulated in oesophageal cancer by a different group in the same year, who named it FzE3 [95].

Following the discovery of Fzd genes in *Drosophila*, intense research into the identification, expression and function of Wnt and Fzd homologues during development in other model organisms such as *Xenopus*, *C. elgans* and vertebrates (mice) ensued. Indeed, the successful cloning of Xenopus Fzd7 (*Xfz7*) by Wheeler and Hoppler [96] facilitated a surge in the understanding of evolutionary conserved roles for Wnt/Fzd7 signalling during various developmental and adult processes such as neural crest induction [97], gastrulation [98] and intestinal homeostasis [62]. This is particularly well exemplified in the gut. For example, the gastric tube of hydra, which is separated into endodermal and ectodermal layers, express Wnt signalling orthologues such as *β-catenin* [99], *Fzd7*, *Gsk-3β*, *Dvl*, *Apc*, and *Axin* from the Wnt/β-catenin pathway [92,100] and *Van Gogh*, *Flamingo* and *JNK* from the Wnt/PCP pathway [101] along an oral-aboral axis during gastrulation [102]. The organisation and coordinated balance of cell renewal and movement of epithelial and interstitial cells throughout the gastric tube is regulated by Wnt/Fzd signalling [103,104,105]. Of note, when compared to the ten mammalian Fzd family members, the hydra Fzd shares closest homology with Fzd7 (and *Drosophila* Fzd2) [92,106], which suggests an essential role for Fzd7 in regulating cell differentiation, proliferation and the establishment and homeostasis of three-dimensional gut tissue architecture, which has been maintained throughout millions of years of evolution as it is also critical in higher organisms [62].

Indeed, *Xfz7* has been shown to regulate the coordinated convergent extension movement of cells [67], tissue separation [98] and dorsal axis specification [107] during gastrulation in developing *Xenopus laevis*. Furthermore, the absolute requirement for canonical Wnt signalling in the developing and adult intestinal epithelium implicates a pivotal role for Fzd receptors [51,52,108,109]. In the mammalian intestinal epithelium, Fzd7 expression is restricted to the stem cell/progenitor compartment, the crypts of Lieberkühn, suggesting a role in Wnt signal transduction to intestinal stem cells [62,110]. As such, Fzd7 has been recently shown to be the Fzd receptor responsible for transmitting key Wnt signals to intestinal stem cells, facilitating intestinal stem cell maintenance, epithelial homeostasis and regeneration following DNA damage [62].

*Fzd7^−/−^* mice are viable and have a mild truncation and kink in the tail [111], which is reminiscent of the loop-tail phenotype in PCP mutant mice [112,113,114,115], suggesting Fzd7 partially regulates PCP signalling during development. However, as *Fzd7^−/−^* mice do not have an intestinal phenotype, this suggests that PCP signalling via Fzd7 is less important during intestinal development and homeostasis. Furthermore, canonical Wnt signalling is absolutely required during intestinal development and homeostasis [108]. Therefore regulation of canonical Wnt signalling via Fzd7 must be functionally compensated by other frizzled receptors in *Fzd7^−/−^* mice during development and homeostasis of the intestine. Fzd2 has been demonstrated to compensate for loss of Fzd7 during development, since compound *Fzd2^−/−^*; *Fzd7^−/−^* mice die at E10.5, whilst *Fzd1^−/−^*; *Fzd7^−/−^* are healthy and fertile [111]. Although genetic deficiency of *Fzd7* can be tolerated during development and homeostasis of the intestine, surprisingly, acute genetic deletion of *Fzd7* is deleterious in intestinal stem cells [62]. This could be due to a combination of factors.

Firstly, in *Fzd7^−/−^* mice, the compensation mechanism using Fzd2 in the absence of Fzd7, has been occurring all through development and thus the system is fully established in the adult intestinal epithelium. This is a very different scenario from acute, conditional deletion which would result in significant stress for intestinal stem cells, which require Wnt signalling, possibly resulting in a different response from that of continued absence of *Fzd7* from the beginning of conception as in a *Fzd7^−/−^* mouse.

Secondly, conditional deletion of *Fzd7* in the intestinal epithelium triggers rapid repopulation of the epithelium with *Fzd7* proficient cells, which is not possible in a *Fzd7^−/−^* mouse [62]. Conditional deletion of other genes in the intestine, including *c-Myc* [116] and *Chk1* [117], also trigger rapid repopulation, and thus it seems this mechanism is a sufficient safe-guard to protect intestinal stem cells if they experience the deletion of a critical gene such as *Fzd7* [62], rather than relying on Fzd2 to transmit the crucial Wnt signals. Thirdly, the capacity of the intestinal epithelium to regenerate after irradiation-induced DNA damage is markedly reduced in *Fzd7^−/−^* mice, however regeneration does recover and is associated with an increased expression of *Fzd2* and *Fzd1*, suggesting that if repopulation with *Fzd7* proficient cells is not possible (as in a *Fzd7^−/−^* mouse), then compensation with other *Fzd* genes can occur [62].

Fzd7 is able to modulate the transmission of both canonical and non-canonical Wnt pathways. There are many examples of Fzd7 transmitting canonical Wnt signalling in several tissues during development, homeostasis and disease in many organisms including humans [118]. This occurs via Fzd7 associating with Lgr4/5 [55] and a Wnt ligand such as Wnt3, and is greatly enhanced in the presence of R-Spondin which prevents Rnf43 mediated degradation of the Fzd7 receptor complex [119]. In the neural crest, Fzd7/Wn6 induces epithelialisation of the somites via β-catenin [120], while Fzd7/Wnt11 orchestrates neural crest cell migration via the PCP and Ca^2+^ pathways [24].

Another layer of complexity that can influence the activation of specific Wnt/Fzd pathways is the subcellular localisation of Fzd receptors. This was formally demonstrated in experiments overexpressing chimeric DFzd1 and DFzd2 proteins in the *Drosophila* wing imaginal disc epithelium. This study revealed that the intracellular C-terminal of Fzd proteins is responsible for directing the localisation of specific Fzd receptors within an apical-basal axis, which in turn direct either PCP (apical) or canonical (basal) Wnt signalling [121]. Of note, the recent visualisation of a short-range Wnt gradient operating in the intestinal stem cell niche identified Wnt3, a prototypical canonical Wnt ligand, to be expressed on the baso-lateral membrane of Paneth cells, which is received by Fzd receptors on adjacent Lgr5^+^ stem cells [10]. This finding is consistent with observations that Fzd7 is expressed in Lgr5^+^ intestinal stem cells [62,122] on the baso-lateral membrane [123], and is required for their function *in vivo* [62]. It will be of future interest to investigate whether the intracellular C-terminal of Fzd7 is required for its baso-lateral localisation and how this may impact on intestinal stem cell function.

During *Xenopus* development Fzd7 can form a complex with R-Spo3 to transmit Wnt5a signals and activate PCP signalling by inducing Sdc4-dependent, clathrin-mediated endocytosis [60]. These data support a model whereby internalisation of the Wnt/receptor complex is an important mechanism by which R-Spondins promote Wnt signalling.

Fzd7 can also co-operate with Ryk co-receptors to transmit Wnt 11-mediated signals via β-arrestin2-dependant endocytosis and JNK in convergent extension during *Xenopus* development [67]. Furthermore, low levels of β-catenin and non-canonical JNK (PCP) signalling can both be activated by Fzd7 to regulate foregut fate and proliferation during *Xenopus* development [124]. Fzd7 has also been shown to regulate PKC, and its translocation to the cell membrane during *Xenopus* gastrulation, suggesting it can regulate the Ca2+ pathway [98]. Recently it has also been demonstrated that Fzd7 (and Fzd1, Fzd2 and Fzd6) is able to directly interact with G proteins to activate MAPK (PCP) signalling [71].

Finally, the specification and expansion of human embryonic stem cells (hESCs) during development relies on controlled spatial and temporal Wnt signalling. Meta-analysis of 38 publications studying gene signatures of hESCs identified that FZD7 is consistently expressed highlighting that FZD7 is the predominant FZ receptor in many stem cell populations [125]. This was also observed at the protein and transcriptome level [126]. Indeed, canonical Wnt signalling via Wnt3a and FZD7 promote the proliferation of hESCs and subsequent knockdown of FZD7 has been shown to induce hESC differentiation [127,128]. However, interestingly, in the same model system, non-canonical Wnt signalling activated by Wnt11 and FZD7, is sufficient to direct hESCs from pluripotency towards mesodermal lineage specification [23]. Collectively, these studies demonstrate not only the promiscuity of Wnt/Fzd interactions, but how subtle changes in the availability of Wnt ligands and/or receptor components can direct different cellular outcomes.

The Wnt-Fzd interaction is promiscuous, with a single Wnt able to bind multiple Fzd proteins, and vice versa, to activate canonical and/or non-canonical Wnt signalling. Successful crystallisation of *Xenopus* Wnt8 bound to Fzd8 CRD revealed two distinct residue moieties that facilitate Wnt/Fzd binding [129]. One is via a palmitoleic acid lipid group found on the Wnt protein that projects into a deep groove in the Fzd8 CRD. The second is a conserved finger-like projection that forms a hydrophobic amino acid interface with a depression in the opposite side of the Fzd8 CRD [129]. The authors of this important work also conclude that the binding chemistry of the Wnt/Fzd interaction strongly suggests that mono-specificity is not compatible and thus it is highly unlikely that a restricted ligand-receptor matched code exists [129].

The Wnt-Fzd structure reveals the precision with which Wnt binds Fzd and provides evidence for complex oligomerisation with co-receptors to transduce signals to cytoplasmic signal transducers, most notably, Dvl [129]. The Fzd receptors are thought to homo- or hetero-dimerise in order to signal, which adds another level of complexity to Wnt/Fzd signalling [40,129]. This notion, based on the crystal structure of the CRD region, is supported by Fzd oligomerisation assays, which show that each Fzd tested (Fzd1, 2, 4, 7, 9) homo-oligomerise, and that some Fzd receptors are able to hetero-oligomerise (e.g., Fzd1, 2 & 7). Notably, Fzd7 could oligomerise with each Fzd tested [65], and is also able to associate with several co-receptors which may help explain why Fzd7 is the only member of the Fzd family that has been demonstrated to activate each branch of the pathway: Wnt/β-catenin pathway [107,130], the PCP pathway [22,131] and the Wnt/Ca^2+^ pathway [98].

This promiscuity of Fzd7 to associate with different co-receptors and regulate both arms of the Wnt pathway may explain its frequent up-regulation in many different cancers to drive tumour growth, as both pathways are implicated in cancer. Although a variety of proteins have been discovered to bind Fzd7, not all of them have been functionally characterised [70] (Table 1 listing the different proteins that Fzd7 can bind to and the signalling output). However Fzd7 is not unique in its ability to activate canonical and non-canonical pathways, with examples also published for other Fzd’s. For example, Fzd5/Wnt5a activates the Ca^2+^ pathway in melanoma and is associated with a more aggressive phenotype [132], whilst in the developing retina Fzd5 signals via β-catenin [133].

These exciting developments have spawned a new focus in cancer research to target the Wnt ligand/receptor complex as a potentially therapeutic strategy to treat several different cancers detailed in the sections below.

### Regulation of Fzd7

Gene regulation is governed by a complex network of cellular mechanisms, which can be modulated at every step. As such, the ever-expanding interplay between signaling pathways, transcription factors and post-transcriptional modifiers demonstrates that the expression of a single gene is not exclusively governed by a single factor. Indeed several recent studies have demonstrated the expression of *Fzd7* to be regulated by multiple highly conserved signalling pathways and post-transcriptional modifiers (miRNAs). Wnt/β-catenin signaling is one of the key signalling pathways shown to regulate *FZD7* expression, as *FZD7* expression is increased in human embryonic carcinoma cells following exposure to Wnt3a conditioned medium [87]. In addition, the *FZD7* promoter houses putative TCF binding sites, confirming its regulation by Wnt/β-catenin signaling [87]. Wnt/β-catenin-mediated regulation of *FZD7* is further supported by findings demonstrating the activation and repression of a *FZD7* promoter-driven luciferase reporter in response to β-catenin stimulation and inhibition respectively [88]. However, given the increasing complexity of crosstalk between signalling pathways, it’s of little surprise that *Fzd7* expression is regulated by more than just Wnt signalling. For instance, unpublished data from our own lab indicates that *Fzd7* is a transcriptional target of Jak/Stat3 signalling [123]. These findings are corroborated by observations made in articular chondrocytes, demonstrating a reduction in *FZD7* expression following pharmacological inhibition (AG490) of JAK2 [134]. More recently, the Notch signaling pathway, specifically *NOTCH3*, was shown to regulate the expression of *FZD7* in human mammary epithelial cells [135]. Further analysis revealed *FZD7* to be expressed predominantly by luminal progenitor cells, which is hypothesised to regulate luminal cell differentiation through a *NOTCH3-FZD7* signalling axis [135].

Furthermore, microRNAs (miRNAs or miRs), small non-coding RNAs that influence post-transcriptional gene expression through imperfect binding to the 3’ UTR of mRNA, play important roles in development and disease, placing them as potential therapeutic targets. As such, several miRNAs have been identified to influence the activation of Wnt signalling via regulation of *Fzd7* expression. For instance, miRNA-206 is a critical myogenic regulation factor required for correct skeletal muscle development and differentiation [136]. Subsequent algorithmic and bioinformatic analysis of gene expression databases reveal *Fzd7* to be a direct target of miRNA-206 [137]. As such, overexpression of *Fzd7* induces increased proliferation and abnormal myoblast differentiation [137]. These findings are supported by previous studies demonstrating a central role for Fzd7 in skeletal stem cell maintenance in muscular dystrophy animal models via activation of Akt/mTOR signalling [21].

Wnt signalling is a critical signaling pathway during the development, homeostasis and regeneration of the intestinal epithelium [8,62,138]. Recent research has identified several miRNAs, including miRNA-222, that influence intestinal epithelial biology [139]. Intestinal regeneration was significantly impaired in a novel miRNA-222 overexpressing transgenic mouse, which was associated with impaired Wnt activation, through downregulation of *Fzd7* [140]. Indeed, Fzd7 was subsequently shown to be a direct target of miRNA-222, thus revealing induction of miRNA-222 negatively regulates intestinal regeneration through suppressing the expression of *Fzd7* [140]. These data are strongly supported by recent work showing intestinal regeneration to be significantly impaired in *Fzd7^−/−^* mice [62].

Likewise, miRNAs have been shown to influence the expression of *Fzd7* in several different cancers, and associated with reduced tumour growth, which are discussed in the relevant cancer sections below. However, recently Li *et al.* uncovered a two-faceted role for miRNA-126 in acute myeloid leukemia (AML), demonstrating that either overexpression or knockout of miRNA-126 could promote AML via inducing the expression of oncogenic fusion genes [141]. Interestingly, knockdown of miRNA-126 repressed the expression of tumour suppressor genes *ERRFI1* and *SPRED1*, but increased Wnt pathway activation via increased *FZD7* expression. This resulted in accelerated AML progession, however, cells were sensitised to standard chemotherapy agents [141]. Collectively, these studies reveal some of the factors regulating *Fzd7* expression, and illustrate its importance in several biological contexts, from development and regeneration through to homeostasis and cancer.

## 7. Intestinal Cancer

Wnt signalling has been recognised as an important driver of intestinal cancer for many years. Mutations to *APC* were first observed in colon tumours of FAP (familial adenomatous polyposis) patients in the 1990’s [142,143,144]. Subsequent studies demonstrated that Apc is a potent tumour suppressor; a mutagenesis screen in mice identified that mutation to *Apc* caused multiple intestinal adenoma’s (these mice were called Apc^Min/+^) [75,145], whilst conditional truncation of *Apc* throughout the intestine using transgenic mice resulted in rapid uncontrolled proliferation similar to tumourigenesis [146,147]. Furthermore, intestinal stem cells, marked by the Wnt target gene *Lgr5* were identified as the cell of origin for intestinal cancer, as truncation of *Apc* specifically in Lgr5+ cells resulted in widespread, rapid, development of adenoma’s throughout the small intestine and colon [148]. Although *β-catenin* mutations are observed at much lower frequencies than *Apc* in intestinal tumours (~5% compared to ~75%) [3], conditional activation of β-catenin is sufficient to induce tumourigenesis in the mouse intestine [149,150], supporting the model of cytoplasmic Wnt signalling components as regulators of intestinal cancer. Indeed, truncating mutations in *Apc* or oncogenic mutation in *β-catenin* are mutually exclusive but appear to be obligatory initiating steps for CRC [151]. Thus, intestinal tumourigenesis requires constitutive activation of the canonical Wnt pathway, although the aetiology of APC and β-catenin mediated intestinal cancer is different [151,152]. Oncogenic mutations in *β-catenin* are more common in intestinal tumours with microsatellite instability [3]. Intriguingly, *APC* truncation may provide the genetic make-up for additional regulation by the Wnt pathway (reviewed in [106] and papers cited within). For example, LEF-1 over-expression alone is sufficient to induce EMT in a developmental context, but both LEF-1 over-expression and APC truncation are required to induce EMT in a colon cancer setting. Furthermore, restoring full length APC is sufficient to abrogate tumour growth in human colon cancer cells *in vitro* [153], or using a mouse model which allows deletion and restoration of Apc *in vivo* [154].

Due to the high frequency of Apc mutations observed in intestinal cancer it was assumed that the Wnt receptor complex would not be important in regulating Wnt activity in this setting. However, as described above, SFRPs, WIF-1 and DKK1 are often hypermethylated and downregulated very early in intestinal cancer suggesting the Wnt receptor complex does play a role [79,80,155]. Indeed the Wnt agonists RSPO2 and RSPO3 are fused in 10% of human colon tumours and act to potentiate Wnt signalling [156]. As such, the treatment of human patient-derived colon tumour xenografts with specific monoclonal antibodies generated against RSPO family members (anti-RSPO) have shown promising growth inhibition and reduced Wnt/β-catenin signalling when used in combination with standard chemotherapy [157]. In addition, loss-of-function (LOF) mutations to the E3 family ligases ZNRF3 and RNF43, which serve to negatively regulate Fzd receptor turnover, are commonly observed in human colon tumour biopsies [3]. Such tumours are predicted to be hypersensitive to Wnt signalling through the stabilisation of Fzd receptors [58].

Several FZD receptors are over expressed in human intestinal tumours and cell lines, including FZD3, FZD6 and FZD7 [1,106,158]. Inhibition of FZD7 using a dominant negative extracellular domain is able to block the growth of human colon cancer cells *in vitro* and in xenograft experiments with stably transfected SK-CO-1 cells, which harbour mutations to *APC* [84]. Recently Fzd7 has also been demonstrated to be the predominant Wnt receptor required to transmit Wnt signalling (via Wnt 3 or 2b) in intestinal Lgr5+ stem cells during homeostasis and regeneration [62]. Conditional deletion of *Fzd7* in intestinal organoids resulted in crypt atrophy and organoid death, whilst conditional deletion of *Fzd7* specifically in the Lgr5+ intestinal stem cells triggered rapid epithelial repopulation in transgenic mice [62], demonstrating that Fzd7 is critical for intestinal stem cell function. As Lgr5+ stem cells are the cell of origin for intestinal cancer and also a marker of intestinal cancer stem cells, this data opens new avenues of research to determine the therapeutic benefit of inhibiting Fzd7 to specifically target these cells.

In addition to its role in the very first stages of tumourigenesis, Wnt signalling is also implicated in the progression and invasion of intestinal tumours. Analysis of gene expression in the metastatic potential of human primary colon cancers identified several Wnt target genes to be important, including BAMBI, BOP1, CKS2 and NFIL3. Inhibition of these Wnt target genes with shRNA was able to reduce migration and invasion *in vitro* and also completely block the formation of secondary liver tumours in mice following intrasplenic injection of SW620 cells transduced with the corresponding shRNA [159].

Fzd7 also plays an important role during metastasis by regulating the critical stages in which epithelial cancer cells transition into mesenchymal cells when leaving the primary tumour (EMT), and then from mesenchymal to epithelial (MET) at the secondary site [152,160,161,162,163]. Expression of a dominant negative FZD7 in human SK-CO-1 cells blocked their growth in xenograft experiments and induced morphological changes suggesting that FZD7 promotes MET [84]. The role of Fzd7 during MET was further supported in experiments using a novel model of tumour morphogenesis termed LIM1836-*Mph* cells (human colon cancer cells) which go through cycles of EMT and MET *in vitro* [158]. MET in these cells is associated with a sharp increase in nuclear β-catenin, and treatment with FZD7 specific shRNA is able to inhibit MET suggesting FZD7 regulates canonical Wnt signalling in this process [158]. On the other hand, nuclear β-catenin was very low in migrating LIM1836-*Mph* cells, however FZD7 shRNA could also inhibit migration of these cells suggesting Fzd7 mediates non-canonical signalling of migrating colon cancer cells [158]. Indeed, this was subsequently formally demonstrated by Ueno and colleagues who showed FZD7-mediated vertebrate PCP pathway is involved in colon cancer cell migration [164].

Furthermore, high-throughput screening for miRNAs implicated in human carcinomas has revealed significant downregulation of miR-23b in human colon cancers, which promotes metastasis, tumour growth, invasion and angiogenesis [165]. Subsequent investigations demonstrate that FZD7 is a direct regulatory target of miR-23b and targeted inhibition of FZD7 or miR-23b can significantly reduce migration, growth and angiogenesis in human colon cancer cells [165].

## 8. Hepatocellular Carcinoma

By far the most characterised cancer with relation to FZD7 is Hepatocellular carcinoma (HCC). Between 40%–70% of HCC display elevated nuclear β-catenin [166] suggesting that canonical Wnt signalling maybe important, and it is especially high in HCC associated with Hepatitis B virus (HBV) infection [167]. Although intracellular components of the Wnt pathway such as Axin1 and β-catenin are mutated in some cases of HCC, they are not sufficient in number to account for the high frequency of Wnt pathway activation observed, suggesting activation of the pathway at the level of the ligand/receptor. Indeed, several Wnt ligands are overexpressed in HCC, including Wnt1/3/4/5a and 10b, whilst Wnt antagonists sFRP1/4/5, Wif1, Dkk3, Dkk4 are frequently reduced [168]. Many of these genes have been functionally demonstrated to regulate tumour growth, thus highlighting that HCC can be modulated by Wnt signalling at the level of the receptor [168]. Indeed, serum levels of Dkk1 can be used to diagnose HCC and differentiate HCC from HBV infection [169].

FZD7 is frequently over expressed in HCC, especially in patients infected with HBV [167], and has been shown to interact with Wnt3a to transmit canonical Wnt signalling in HCC [63]. One strategy to treat HCC via manipulation of FZD7 may involve the use of soluble peptide fragments to antagonise FZD7 signalling. For instance, HCC cell lines (HepG2 and Huh7) treated with a recombinant soluble FZD7 (sFZD7) peptide, which contains the extracellular domain of FZD7, inhibits Wnt/β-catenin signalling and decreases cell proliferation and viability (without effecting normal hepatocytes) [170]. The effects of sFzd7 on abating HCC tumourigenicity were accomplished by the competitive binding of the Fzd7 extracellular CRD with Wnt3, which activates the canonical Wnt signalling pathway [170]. This is consistent with results observed in colon cancer cells using the same strategy [84].

Post transcriptional control of FZD7 is also observed in HCC. The expression of miR199a is frequently downregulated in human liver tumours, and associated with poor clinical outcome. Experimental overexpression of miR199a in human HepG2 HCC cells was able to repress cancer growth *in vitro* and *in vivo*, and was associated with inhibition of FZD7 [171].

FZD7 is overexpressed in the surrounding periportal and dysplastic liver suggesting it may be important during early stages of HCC [63]. However, the precise role of Wnt signalling during the very early stages of the disease are still the subject of debate as conditional activation of Wnt signalling in transgenic mouse models reveals that elevated Wnt signalling causes hepatomegaly and disorganisation of liver zonation, but does not result in the development of tumours [172,173]. It seems therefore that deregulated Wnt signalling acts more to promote HCC in co-operation with other oncogenic pathways such as AKT, MET, H-RAS, insulin receptor substrate-1 and chemical mutagens. This does not make targeting FZD7 for the treatment of HCC any less attractive as mono-therapies have failed to provide any robust clinical outcomes and co-treatments targeting multiple pathways is now the focus of most targeted therapy approaches.

## 9. Gastric Cancer

Wnt signalling is emerging as an important oncogenic pathway in gastric cancer (GC). The recent Cancer Genome Atlas paper, which analysed 295 primary gastric adenocarcinomas, identified that *APC* is the 6^th^ most frequently mutated gene in the non-hypermutated gastric tumours (over three times more frequent than the hypermutated subgroup which does not contain *APC* mutations). When combined with gain-of-function mutations in β-catenin, the Wnt pathway is found to be activated in over 10% of GCs [174]. These observations are consistent with a second paper investigating molecular profiling of GC in *Nature Genetics* in which the authors conclude that aberrant Wnt/β-catenin signalling plays an important role in gastric adenoma [175]. Deregulation of Wnt signalling by conditional deletion of all 4 copies of Gsk3 (both α and β isoforms) was able to induce formation of gastric tumours in mice, thus providing functional proof that activation of Wnt is able to trigger tumourigenesis in the gastric epithelium *in vivo* [176].

Components that are known to be associated with signalling via FZD7 are strongly implicated in GC. For example, it has been shown extracellular secreted Wnt antagonists (sFRP and Dkk) are frequently epigenetically silenced through promoter hyper-methylation [81,177]. Critically, exogenous re-introduction of Wnt pathway inhibitors, such as sFRP1, 2 or 5 and Dkk1, or reversing promoter methylation can significantly limit and reduce *in vitro* tumourgenicity and tumour xenograft burden of Wnt pathway-activated gastric cancer models by means of attenuating Wnt/β-catenin signalling [81,177,178]. Importantly, these studies demonstrate (a) activated Wnt signalling is sufficient to drive gastric tumourigenesis and (b) provide proof-of-principle that modulation of extracellular/upstream signalling components can impact on Wnt pathway output irrespective of intracellular/downstream mutations that would otherwise drive positive feed-forward Wnt signalling. Furthermore, the fact that sFRPs and Dkks act by inhibiting Wnt/FZD signalling implies a role for Wnt/FZD in tumourigenesis of GC.

There are several reports suggesting that the non-canonical Wnt pathway is active in GC. RHOA, a downstream component of non-canonical Wnt signalling and FZD7 [179], is upregulated in GC [174,180], whilst ROR1, a tyrosine kinase like receptor able to transmit non-canonical Wnt5a signals [181], is phosphorylated in HS746T human gastric cancer cells, via a Met dependant mechanism [31]. Wnt5a, which primarily activates the non-canonical pathway, is up-regulated in human gastric tumours, however, this is not observed in cultured human GC cell lines (OKAJIMA, TMK1, MKN7, MKN28, MKN74 and KATOIII) suggesting the source of Wnt5a is from stromal cells [182]. Similarly, Wnt5a and Wnt2 were found to be significantly up-regulated in colon cancer associated macrophages [183], illustrating that Wnt signals can originate from outside the tumour cells themselves.

Several reports demonstrate that FZD7 expression is upregulated in GC, with high expression associated with poor clinical outcome [184,185,186]. However, mutations in RNF43, an E3 ubiquitin ligase that regulates FZD receptor turnover and therefore activate Wnt signalling, are frequently observed in GC [174]. This data, together with methylation of SFRPs (frequently observed in GC) identifies additional mechanisms that increase Wnt signalling, which cannot be identified by transcriptional and mutational analysis of FZD’s itself, and suggests that the importance of FZD in GC could presently be underestimated. FZD7 expression is also increased in a side population of cells isolated from GC cell lines, which were found to have increased cancer stem cell properties, supporting a role for FZD7 in promoting GC [186]. Indeed, conditional deletion of *Fzd7* is able to inhibit the growth of gastric cancer cells and in mouse models of GC and colony forming assays with human GC cells [123].

Finally, the strong association between aberrant Wnt signaling and *H. pylori*-induced gastric tumourigenesis, implicates a role for Fzd receptors to promote Wnt signalling in gastric cancer. As such, recent research has shown *FZD7* to be highly expressed following *H. pylori* infection and associated increases in cell proliferation [187]. Subsequent analysis demonstrates *FZD7* to be negatively regulated by miRNA-27b, which when overexpressed is sufficient to inhibit the expression of *FZD7* and reduce gastric cancer cell proliferation. Likewise, targeted knockdown (siRNA) of *FZD7* in *H. pylori*-induced gastric cancer cells effectively phenocopies the effect of miRNA-27b overexpression [187].

## 10. Breast Cancer

Wnt signalling regulates the development and homeostasis of the breast [188] and is deregulated in breast cancer. Indeed Wnt1 was first identified as a gene, the ectopic expression of which via pro-viral insertion, was able to promote mammary tumourigenesis [4]. SFRPs and WIF are frequently methylated and consequently down regulated in breast cancer leading to activation of the Wnt pathway [189,190]. Similarly, LRP6 overexpression was found in a subtype of ER-negative and HER2-negative breast tumours [191].

A screen to analyse which pathways were active in triple negative (TN) breast cancer, identified that the Wnt pathway was an important driver in this aggressive type of breast cancer and that FZD7 was often overexpressed [192]. Indeed, knockdown of FZD7 using shRNA was able to inhibit migration, colony formation and xenograft growth of human MBA-MB-231 TNBC cells which was associated with reduced canonical Wnt signalling [192].

Furthermore, recent research has revealed a novel mechanism by which FZD7 expression is regulated in breast cancer cells. SIRT1, an NAD^+^-dependent deacetylase positively regulates FZD7 mRNA and protein levels, promoting increased cell migration and proliferation [193]. As such, genetic and/or pharmacological inhibition of SIRT1 significantly reduced FZD7 levels through perturbing the binding of β-catenin and c-Jun transcription factors to the endogenous FZD7 promoter, reversing the potent tumourigenic effect of FZD7 overexpression in breast cancer cells [193]. In addition, an elegant study from Yibin Kang’s group recently demonstrated a separate regulatory mechanism of FZD7 in human mammary stem cells (MaSCs) and TN breast cancer cells. A specific isoform of the p63 family, ΔNp63, is commonly expressed in the normal mammary epithelium, where it regulates MaSCs and when overexpressed is sufficient to transform normal MaSCs to tumour-initiating cells (TICs) [194]. Gene-set enrichment analysis (GSEA) of *ΔNp63^/+^* MaSCs reveals alterations exclusively to Wnt signalling, with substantial decreases in *Fzd7* and *Wnt5a* expression, resulting in a significant reduction in MaSCs [194]. Chromatin immunoprecipitation (ChIP) analysis on wild type MaSCs demonstrates a strong enrichment of the *FZD7* enhancer in p63 bound chromatin, which was further supported by the activation of a *FZD7* enhancer-driven luciferase reporter in response to *ΔNp63*, thus demonstrating *Fzd7* is a direct target of *ΔNp63* [194]. Subsequent overexpression and knockdown studies targeting *ΔNp63* or *FZD7* in patient-derived xenografts (PDX) from TN breast tumours significantly attenuated tumourigenicity, which was associated with a reduction in the number of TICs [194]. These data demonstrate a pivotal ΔNp63-Fzd7-WNT signalling axis, which may represent a driving force for the initiation and maintenance of TN breast tumours, thus serving as a potential target for therapeutic intervention.

## 11. Ovarian Cancer

As with other cancers, a role for the Wnt signalling pathway at the level of the receptor/ligand has been suggested from studies on the SFRP genes. SFRP1 SFRP2 and SFRP5 are methylated in ovarian cancer [195], and experimental overexpression or knockdown, demonstrated that epigenetic silencing of SFRP5 was related to tumour growth, invasion (via EMT) and chemosensitivity in ovarian cancer [196].

Ovarian cancer is a heterogenous disease but can be broadly separated into five distinct subtypes based on molecular characteristics; epithelial-A (Epi-A), Epi-B, mesenchymal (Mes), Stem-A and Stem-B. FZD7 is upregulated in the Stem-A subgroup of ovarian cancer, which has a poor clinical outcome [197]. Knockdown of FZD7 using siRNA was able to inhibit proliferation of human ovarian adenocarcinoma cell lines [179] associated with changes in cell morphology which have also been observed in colon cancer cells [84].

Interestingly, inhibition of FZD7 in ovarian cancer cells resulted in an increase in the level of canonical signalling, whilst the non-canonical signalling components including RhoA were slightly down regulated. These data suggest FZD7 maybe regulating ovarian cancer cell growth via the Wnt/PCP pathway rather than the canonical pathway, which is upregulated in Stem-A ovarian tumours [179].

## 12. Cervical Cancer

Nuclear β-catenin is observed in ~70% of cervical tumours indicating deregulation of Wnt signalling is common in cervical cancer [198]. However, mutations to *APC* and *β-catenin* are rare in this cancer, suggesting that Wnt signalling could be activated at the level of the receptor in cervical cancer [198]. Indeed, this is supported by studies reporting hypermethylation and downregulation of DKK-3 [199], WIF-1 [200], SFRP-1, SFRP-2 and SFRP-4 [201] in cervical tumours.

Inhibition of FZD7 using shRNA was able substantially reduce invasion and EMT in HeLa and SiHa cervical cancer cell lines *in vitro* [202]. These phenotypes were associated with changes in the regulation of EMT markers including E-Cadherin, Vimetin and Snail, and also down regulation of MMP genes which are implicated in invasion. Levels of phosphorylated c-Jun and JNK were also reduced when FZD7 was inhibited suggesting the PCP pathway maybe involved in transducing signals downstream of FZD7 in cervical cancer, although no assays were performed to determine the activation of canonical Wnt signalling [202]. Moreover, subsequent studies from the same lab demonstrated *FZD7* to be a direct target of miRNA-142-3p, which is normally expressed at low levels in cervical cancer cells. Importantly, restoration of miRNA-142-3p expression significantly reduced *FZD7* expression, cell proliferation and invasion, thus identifying miRNA-142-3p and FZD7 as attractive therapeutic targets for cervical cancer therapy [203].

There is no data to demonstrate that FZD7 is upregulated in cervical cancer, however, as mentioned above, the role of FZD7 in cancer could be underestimated, as seems the case in cervical cancer, due to changes in the membrane turnover of FZD7, which will greatly affect the response of a cell to Wnt ligands.

## 13. Melanoma

There are conflicting reports on the role of canonical Wnt signalling in melanoma. Some reports have shown that canonical Wnt signalling is reduced as melanoma progresses, suggesting that activating canonical Wnt signalling could be a therapeutic strategy for treatment of melanoma. Indeed, overexpression of Wnt3a reduced proliferation of human or mouse melanoma cells [204], and pharmacological activation of Wnt via treatment with Riluzole [205] or a GSK3 inhibitor (LY2090314) inhibits melanoma cell growth [206]. Conversely, it has been shown that cell intrinsic canonical Wnt signalling mediates resistance to recent immunotherapy approaches such as PD-1 inhibitors [207], thus suggesting that activation of Wnt would not be an effective therapy, at least not in combination with the promising immune check point inhibitors. Of note, Wnt5a is able to promote melanoma progression via Fzd5 and the Ca^2+^ pathway [132], and as canonical Wnt signalling has been shown to be able to inhibit non-canonical signalling, this is a potential mechanism that may help explain how increased canonical Wnt signalling results in reduced melanoma growth [208,209].

FZD7 is upregulated in human, metastatic melanoma cells, and shRNA mediated knockdown of FZD7 in these cells was able to inhibit formation of xenograft tumours and also metastasis following intravenous injection [210]. Melanoma’s which develop resistance to treatment with BRAF inhibitor PLX4720 display upregulated Wnt5a which was consequently show to promote tumour growth via FZD7 and AKT [208]. Interestingly, treatment of MA-2 human melanoma cells with WNT3 or LiCl increased canonical WNT signalling, and decreased JNK (non-canonical Wnt signalling) in cells with FZD7 knockdown, suggesting that FZD7 suppresses the canonical Wnt pathway in melanoma cells [132].

## 14. Wilm’s Tumour and Renal Cancer

Wilm’s tumour (WT) is a renal solid tumour, most commonly observed in children. Canonical Wnt signalling is frequently deregulated in Wilm’s tumour, associated with mutations to β-catenin [211], and FZD7 is also frequently overexpressed [212].

Inhibition of FZD7 using a blocking antibody resulted in reduced clonality of cancer cells isolated from human WT samples, and apoptosis in those cells which expressed FZD7. Interestingly, WT cells become resistant to treatment with FZD7 antibody when SFRP1 or DKK1 are expressed, suggesting that the Wnt pathway is deregulated at the level of the Wnt receptor complex in WT [213].

In renal cell carcinoma (RCC), Wnt signalling is also activated, with reports of loss of Apc [214] and mutations to β-catenin [215]. Indeed, conditional truncation of *Apc* in the kidneys of mice resulted in multiple renal tumours, indicating that deregulated canonical Wnt signalling is sufficient to induce tumourigenesis in the adult kidney [216].

Deregulation of Wnt signalling at the level of the Wnt receptor is also implanted in RCC as SFRP1, SFRP2, SFRP4, SFRP5, DKK3, and WIF1 are hypermethylated in primary RCC samples [217]. FZD7 has recently been shown to be upregulated in ~36% of human RCC [218]. Addition of Wnt3a is also sufficient to increase the growth of human RCC cells *in vitro*, whilst inhibition of FZD7 with shRNA is able to reduce proliferation in the human RCC cells [218], suggesting FZD7 may be an attractive therapeutic target to treat RCC. However, the modest reduction in proliferation of RCC cells upon FZD7 inhibition suggests that strategies combining current standard-of-care therapies for RCC with FZD7 inhibition could be an attractive approach rather than mono-therapies which are often unsuccessful.

## 15. Fzd7 in Other Cancers and Tissues

Deregulated Wnt signalling is observed in many cancers, and, as described above, the Wnt receptor complex is often involved in this aberrant Wnt activation. Although the role of FZD7 has not been described in all cancers with high Wnt activity it is tempting to speculate that FZD7 may be involved in these cancers also, particularly as it is a Wnt target gene [87,88], and it will be interesting in future studies to examine to full extent to which FZD7 is required in the following cancers.

*FZD7* is upregulated in esophageal cancer [95], but there is no functional data to determine its role. Likewise, *FZD7* is upregulated in nasopharyngeal carcinoma [219,220], and Wnt signalling is deregulated in head and neck cancer [221], but the importance of FZD7 has not been studied. Wnt signalling is also deregulated in lung cancer [222,223] and prostate cancer [224,225], but it is unknown if FZD7 is important in these two devastating cancers with very high morbidity rates. Developmental pathways are often deregulated in cancers, and thus it is tempting to speculate whether FZD7 may be involved in hematopoietic malignancies as T-cell maturation in the thymus is blocked by expression of the FZD7 ectodomain [226]. *FZD7* is upregulated in human glioblastoma stem cells, and whilst no experiments have yet been performed to specifically address the role of FZD7 in these cells, overexpression of *sFRP1* halts cell cycle and induces apoptosis, suggesting the Wnt receptor complex, and possibly FZD7, are attractive targets to treat glioblastoma [227,228].

Wnt signalling does not act alone in promoting cancer, and is able to co-operate with other pathways, oncogenes and tumour suppressors, including JAK/STAT [229], K-ras [225,230], p53 [216], mTOR [231] and Notch [232]. Future studies to determine the efficacy of combined targeting of these factors (or re-expression in the case of p53) along with FZD7 inhibition will yield important pre-clinical information, which is likely the future of cancer therapy given the failure of mono-therapies to provide a robust clinical outcome.

## 16. Targeting FZD7 for the Treatment of Cancer

The plethora of studies detailed above has established that signalling via Wnt/FZD is epigenetically regulated in many cancers, and therefore offers an attractive target for therapeutic intervention. As FZD receptors belong to a large family of proteins with high levels of promiscuity to several Wnt ligands it was initially thought that targeting a single FZD receptor would not be an effective strategy to treat cancer due to functional redundancy by another FZD receptor. However, this has not proven to be the case, as experiments inhibiting FZD7 alone have shown that it can have potent anti-tumour effects, as detailed above. Interestingly, DNA damage induced regeneration in the intestine was impaired when *Fzd7* was deleted, but eventually recovered and was associated with an increase in the expression of *Fzd2*, suggesting Fzd2 may have been able to compensate for Fzd7 loss in this setting [62]. It will be interesting in future research to investigate if inhibiting multiple Fzd receptors is able to increase the anti-tumour effects of targeting Fzd7 by eliminating the redundancy between receptors.

Deregulated Wnt signalling in cancer is often the result of mutations to cytoplasmic components of the Wnt pathway. Although the concept of targeting a receptor for a cancer driven by a mutation to a downstream, cytoplasm signal transducer (such as Apc), seems counter-intuitive, the Goldilocks model of Wnt signalling helps explain how this is possible. The data that has contributed to this model demonstrates that mutations to *Apc* do not completely prevent its action to regulate β-catenin and thus upstream events at the level of the receptor can also be targeted in cancers with mutant *Apc* [74]. *Apc* is mutated at several different places, with different levels of Wnt pathway activity associated with each mutation, most likely as a result of the mutations ability to prevent Apc regulating β-catenin phosphorylation. Thus, all *Apc* mutations do not result in the same impact on Wnt signalling, and it will be useful in future research to map which *Apc* mutations correlate to a cellular response to upstream regulators at the level of the ligand/receptor. This information will be important to help stratify which patients will respond to cancer treatments targeting FZD7 or any other regulators of the ligand/receptor complex, based on which mutations to *Apc* their individual cancers have.

Stem cells are required for the continuous tissue maintenance within diverse organs, and Wnt signalling has been identified as regulating stem cells in several organs including the gastrointestinal tract, breast, skin, kidney, and ovary [8]. Stem cells are able to self-renew and proliferate autonomously, if they are located in their niche environment within a tissue, and thus already possess some characteristics of cancer cells [233]. Consequently, stem cells have been identified as the cells of origin for several different cancers including the intestine, stomach, prostate and lung [234]. Wnt signalling has been shown to regulate stem cells in several organs and as such, deregulated Wnt signalling in stem cells is able to induce tumourigenesis in the intestine and stomach [148]. Fzd7 has recently been demonstrated to be the predominant receptor transmitting critical Wnt signals to Lgr5+ intestinal stem cells [62]. Although deletion of *Fzd7* was deleterious to intestinal stem cells, the intestine has a remarkable capacity to repopulate the epithelium with non-recombined, Fzd7 proficient cells, and therefore inhibition of Fzd7 is well tolerated. Thus, future tumour prevention strategies could employ inhibition of FZD7 to prevent Wnt induced transformation of stem cells in FAP patients who are inherently predisposed to develop this type of pathology due to loss of heterozygosity of *APC*. Cancer stem cells are biologically distinct from the differentiated cancer cells that form the bulk of a tumour, and are often the cells responsible for the continued proliferation and resistance to therapies of the tumour [235]. Lgr5 also marks cancer stem cells as well as normal stem cells in intestinal tumours, suggesting FZD7 may offer a novel target to specifically kill cancer stem cells in established tumours. Notably, *FZD7*, but not *FZD2* or *FZD1*, expression is dramatically up-regulated in CRC [158], thus compensation by closely related FZD’s is unlikely. Recently, FZD7+ cells isolated from Wilm’s tumour were shown to be highly clonogenic and proliferative, displaying many stem like gene expression patterns and characteristics, and thus inhibition of FZD7 may also be an attractive strategy to specifically target the cancer stem cells in Wilm’s tumour [213].

Another potential strategy for the use of FZD7 inhibition could be to target disseminated metastatic cells that are responsible for the development of secondary tumours, often many years after the primary tumour has been successfully removed by surgery/chemotherapy. Dormant, disseminated tumour cells are the hindrance to curative treatment for colon cancer, the vast majority of deaths from colon cancer attributable to tumour recurrence. CRC can metastasise early, long before diagnosis, and the disseminated cells can sit dormant for many years, providing ample opportunity to target these cells and eliminate them [152]. As detailed above, FZD7 is able to orchestrate MET in colon cancer cells which is required for disseminated mesenchymal tumour cells to re-establish a tumour at the secondary site, therefore, inhibition of FZD7 could prevent these cells from forming metastases at the new site.

As FZD7 is located on the cell surface, this enables it to be targeted efficiently using antibodies. Indeed, monoclonal antibodies to target FZD7 developed by Oncomed Pharmaceuticals (OMP-18R5 or Vantictumab) are already in phase 1b clinical trials for solid tumours including colorectal, neuroendocrine, breast, sarcoma, pancreatic and NSCLC [236]. As Wnt signalling is critical for the homeostasis of adult tissues, targeting the Wnt pathway for the treatment of cancer has been proved very challenging due to toxicity, mainly to the GI tract and bones [237]. Indeed, the initial enthusiasm for results using Vantictumab were diminished when clinical trials were transiently halted due to bone related side-effects in ~13% of patients. However, a review of the program and subsequent revised safety plan has allowed the trial to continue with a more stringent exclusion plan and bone turn over successfully managed through careful monitoring, and administration of prophylactic Vitamin D, calcium carbonate and zoledronic acid (clinicaltrials.gov NCT02005315). Although Vantictumab was identified in a screen for antibodies that bind to FZD7, it also binds with high affinity to several other FZD receptors including FZD1, 2, 5 and 8 [236]. This may provide for a more potent anti-tumour effect as it negates the redundancy between FZD receptors but may also facilitate toxicity. Future therapies targeting a single FZD are an obvious solution to this but the high levels of sequence similarity, particularly in the N-terminal portion, which would be targeted extracellularly, make this a challenging prospect. To date there are no antibodies which block a specific frizzled receptor. However, genetic evidence demonstrates that deletion of *Fzd7* is able to significantly reduce the transcription of Wnt target genes suggesting that such an antibody would be effective in combating specific cancers [62].

Over recent years it has become clear that activation of the Wnt pathway at the level of the receptor, via upregulation of Wnt ligands and downregulation of Wnt receptor antagonists, plays a major role in promoting cancer initiation, growth and metastasis. Even cancers which contain mutations to cytoplamsic transduction components such as *APC* can respond to FZD7 inhibition. The mechanism for this is not yet fully understood, but could be dependent on the type of mutation present in *Apc*, or the ability of FZD7 to transmit signalling via all the currently known Wnt pathways and consequently by-pass Apc. Thus, FZD7 acts as the lynchpin for multiple Wnt transduction pathways, depending on the availability of co-receptors, ligands and antagonists. This promiscuity of FZD7, may represent the Achilles’ heel of targeting the Wnt receptor complex for the treatment of cancer, as FZD7 is involved in multiple malignancies characterised by deregulated Wnt signalling.

## Figures and Tables

**Table 1:** List of proteins which are shown to associate with Fzd7.

Protein	Tissue/Cells	Interaction Interface	Biological Process	Wnt Pathway Activation	Reference
Wg	293T cells	CRD	Segment polarity	β-catenin	[61]
Wnt2b	293T cells	CRD	Intestinal stem cell niche	β-catenin	[62]
Wnt3a	293T, Huh7 & HepG2 cells	CRD	Intestinal stem cell niche, HCC cell proliferation and migration	β-catenin	[62,63]
Wnt5a	Mouse fibroblasts (L cells)	CRD	Cell migration	PCP & Calcium	[20]
Wnt7a	Skeletal muscle	CRD	Satellite stem cell maintenance	PCP	[21]
Wnt8b	Xenopus	CRD	Xenopus development	β-catenin & PCP	[22]
Wnt9b	Embryonic chicken liver	CRD	Hepatocyte morphogenesis and maturation	β-catenin	[64]
Wnt11	Human embryonic stem cells, embryonic chick	CRD	Mesoderm lineage specification, neural crest migration	PCP & Calcium	[23,24]
Fzd1	293T cells	7TM domain	Oligomerisation in ER	Not determined	[65]
Fzd2	293T cells	7TM domain	Oligomerisation in ER	Not determined	[65]
Fzd4	293T cells	7TM domain	Oligomerisation in ER	Not determined	[65]
Fzd7	293T cells	7TM domain	Oligomerisation in ER	Not determined	[65]
Fzd9	293T cells	7TM domain	Oligomerisation in ER	Not determined	[65]
R-Spo3	Xenopus	Co-localise	Receptor complex endocytosis during Gastrulation	PCP	[60]
Ror2	L cells	CRD	Cell migration	PCP	[20]
Lgr4	LS174T cells	Co-localise	Wnt signal potentiation	β-catenin	[55]
sFRP1	CHO cells	CRD	Cell migration	PCP	[66]
Ryk	Xenopus	Co-localise	Convergent extension	PCP	[67]
Sdc4	Xenopus	Co-localise	Receptor complex endocytosis during Gastrulation	PCP	[60]
PSD-95	Yeast two-hybrid screen	PDZ domain	Not determined	Not determined	[68]
Magi3	Yeast two-hybrid screen, HEK293 cells	C-terminal	Not determined	PCP	[69]
Sap97	Yeast two-hybrid screen	C-terminal	Not determined	Not determined	[68]
Dvl	Xenopus	C-terminal (PDZ domain)	Convergent extension	β-catenin & PCP	[20,22,67]
PI3K	Skeletal muscle	Not determined	Satellite stem cell expansion and myofibre hypertrophy	PCP	[21]
PKN1	293T & A375 cells	Plasma membrane	Inhibits β-catenin phosphorylation	β-catenin	[70]
G-Proteins (Gpa1/Gα_s_)	Yeast two-hybrid screen	C-terminal	Not determined	MAPK	[71]

## Abbreviations

The following abbreviations are used in this manuscript:

Fzd	Frizzled
CRD	Cysteine rich domain
PDZ	amalgam of Post-synaptic density protein 95, Disc-large tumour suppressor and Zonula occludens
MMTV	Mouse Mammary tumour virus
Wg	Wingless
GSK3	Glycogen synthase kinase-3
TCF4	T-cell factor-4
LEF1	Lymphoid enhancer binding factor-1
Apc	Adenomatous polyposis coli
CK1	Casein kinase 1
Lrp5/6	Low-density lipoprotein receptor-related protein 5 or 6
Dvl	Dishevelled
PCP	Planar Cell Polarity
ROR2	Receptor tyrosine kinase-like orphan receptor 2
Ryk	Receptor-like tyrosine kinase
JNK	c-JUN N-terminal Kinase
PKC	Protein kinase C
CaM kinase	Calcium/calmodulin-dependent protein kinase
RhoA	Ras homolog family member A
Rac1	Ras-related C3 botulinum toxin substrate 1
Celsr	Cadherin,EGF LAG seven-pass G-type receptor
Vangl	Vang-like (“Van Gogh”)
IP3	Inositol 1,4,5-triphosphate
Dag	1,2 diacylglycerol
PLC	Phospholipase C
CaMKII	Calcium calmodulin dependent protein kinase II (CaMKII)
NFκB	Nuclear factor kappa-light-chain-enhancer of B cells
CREB	cAMP response element-binding protein
sFRP	Secreted Frizzled Related Proteins
Dkk	Dickkopf
NTR	Netrin realted motif
R-Spo	R-Spondin
Lgr4/5	Leucine-rich repeat-containing G-protein coupled receptor 4 or 5
ZNRF3	Zinc-ring finger 3
RNF43	Ring finger 43
Sdc4	Syndecan-4
PKN1	Protein kinase N1
WIF1	Wnt inhibitory factor 1
MAPK	Mitogen activated protein kinase
hESCs	Human embryonic stem cells
FAP	Familial Adenomatous Polyposis
CRC	Colorectal Cancer
EMT	Epithelial-Mesenchymal transition
MET	Mesenchymal-Epithelial transition
HCC	Hepatocellular Carcinoma
HBV	Hepatitis B Virus
GC	Gastric cancer
WT	Wilms Tumour
RCC	Renal Cell Carcinoma
mTOR	Mammalian target of Rapamycin
JAK	Janus kinase
STAT	Signal Transducer and Activator of Transcription
